# Efficacy of Maternal Influenza Vaccination Against All-Cause Lower Respiratory Tract Infection Hospitalizations in Young Infants: Results From a Randomized Controlled Trial

**DOI:** 10.1093/cid/cix497

**Published:** 2017-05-29

**Authors:** Marta C Nunes, Clare L Cutland, Stephanie Jones, Sarah Downs, Adriana Weinberg, Justin R Ortiz, Kathleen M Neuzil, Eric A F Simões, Keith P Klugman, Shabir A Madhi

**Affiliations:** 1 Department of Science and Technology/National Research Foundation, Vaccine Preventable Diseases, and; 2 Medical Research Council, Respiratory and Meningeal Pathogens Research Unit, University of the Witwatersrand, Johannesburg, South Africa;; 3 Department of Pediatrics, Medicine and Pathology, University of Colorado Denver, Aurora;; 4 Department of Medicine and Department of Global Health, University of Washington, Seattle;; 5 Center for Vaccine Development, University of Maryland, Baltimore;; 6 Department of Pediatrics, University of Colorado School of Medicine and Center for Global Health, Colorado School of Public Health, Aurora;; 7 School of Pathology, University of the Witwatersrand, and; 8 National Institute for Communicable Diseases, National Health Laboratory Service, Centre for Vaccines and Immunology, Johannesburg, South Africa

**Keywords:** influenza vaccine, efficacy, phase 3 trial, lower respiratory tract infections, hospitalizations

## Abstract

**Background:**

Influenza immunization of pregnant women protects their young infants against laboratory-confirmed influenza infection. Influenza infection might predispose to subsequent bacterial infections that cause severe pneumonia. In a secondary analysis of a randomized clinical trial (RCT), we evaluated the effect of maternal vaccination on infant hospitalizations for all-cause acute lower respiratory tract infection (ALRI).

**Methods:**

Infants born to women who participated in a double-blind placebo-controlled RCT in 2011 and 2012 on the efficacy of trivalent inactivated influenza vaccine (IIV) during pregnancy were followed during the first 6 months of life.

**Results:**

The study included 1026 infants born to IIV recipients and 1023 born to placebo recipients. There were 52 ALRI hospitalizations (median age, 72 days). The incidence (per 1000 infant-months) of ALRI hospitalizations was lower in infants born to IIV recipients (3.4 [95% confidence interval {CI}, 2.2–5.4]; 19 cases) compared with placebo recipients (6.0 [95% CI, 4.3–8.5]; 33 cases) with a vaccine efficacy of 43.1% (*P* = .050). Thirty of the ALRI hospitalizations occurred during the first 90 days of life, 9 in the IIV group (3.0 [95% CI, 1.6–5.9]) and 21 in the placebo group (7.2 [95% CI, 4.7–11.0]) (incidence rate ratio, 0.43 [95% CI, .19–.93]) for a vaccine efficacy of 57.5% (*P* = .032). The incidence of ALRI hospitalizations was similar in the IIV and placebo group for infants >3 months of age. Forty-four of the hospitalized infants were tested for influenza virus infection and 1 tested positive.

**Conclusions:**

Using an RCT as a vaccine probe, influenza vaccination during pregnancy decreased all-cause ALRI hospitalization during the first 3 months of life, suggesting possible protection against subsequent bacterial infections that influenza infection might predispose to.

**Clinical Trial Registration:**

NCT01306669.

## Summary

Influenza vaccination during pregnancy decreased the incidence of acute lower respiratory tract infection hospitalizations in infants born to vaccinated mothers. The benefits of protecting against influenza virus infection during early infancy might extend beyond protecting only against influenza-confirmed illness.

Acute lower respiratory infections (ALRIs), such as pneumonia and bronchiolitis, are important causes of morbidity and mortality in children. Although the incidence of childhood ALRI mortality has declined in the past decade, an estimated 0.9 million children younger than 5 years died from pneumonia in 2015, including 0.5 million child pneumonia deaths in sub-Saharan Africa [1]. Furthermore, there were approximately 15 million episodes of under-5 childhood pneumonia hospitalizations in 2010, with the incidence of hospitalization being >3 times higher in newborns and almost 1.3 times higher in the 0- to 11-month age group compared with the overall rate in children 0–59 months of age [2].

Temporal associations between influenza virus circulation and pneumonia hospitalization, which might be due to primary viral or secondary bacterial pneumonia, have been reported [3–5]. A large study in the United States compared the rates of hospitalization for acute cardiopulmonary conditions during influenza seasons to noninfluenza winter time periods and estimated the average annual hospitalization rate attributable to influenza to be highest for infants <6 months of age (104 hospitalizations/10000 children), compared with children 6–12 months and 1–3 years of age (50/10000 and 19/10000, respectively) [6]. Several lines of evidence support a pathogenic synergism between influenza virus and respiratory bacteria. Influenza infections in children increases the risk of new *Streptococcus pneumoniae* serotype acquisition; and because nasopharyngeal acquisition of a new serotype increases the risk of pneumococcal diseases, influenza infections may therefore predispose to the development of bacterial diseases [7, 8].

While active influenza vaccination is the most efficient way to prevent influenza virus infection, current vaccines are poorly immunogenic and not licensed for use in infants <6 months of age. An alternative strategy to prevent influenza illness in young infants is vaccination of pregnant women to achieve passive protection through transplacental transfer of antibodies [9–11]. Vaccination of pregnant women with inactivated influenza vaccine (IIV) is immunogenic and protects the women and their infants against influenza illness [9–11]. Recently we also reported that efficacy of IIV vaccination during pregnancy on preventing polymerase chain reaction (PCR)–confirmed influenza infection in the infants was 86% during the first 8 weeks of life, which decreased to 49% if considering the overall 6-month follow-up period, corresponding to the reduction in maternally acquired influenza antibodies in the infants [12].

Population-based and case-control studies on the effect of influenza vaccination during pregnancy on more severe outcomes in infants, including hospitalizations, have reported conflicting results [13–19]. The objective of this post hoc analysis of a randomized, placebo-controlled trial was to probe the association of influenza vaccination of human immunodeficiency virus (HIV)–uninfected pregnant women in preventing ALRI hospitalization in their infants <6 months of age [10]. The present analysis was restricted to infants born to women enrolled in the HIV-uninfected cohort.

## SUBJECTS AND METHODS

### Study Design

Details of the study have been published [10]. In brief, we conducted a randomized, double-blind, placebo-controlled trial of IIV in HIV-uninfected pregnant women in Soweto, South Africa. This involved 2 cohorts of HIV-uninfected pregnant women in their second/third trimester who were enrolled from 3 March to 4 August 2011 (n = 1060) and 6 March to 2 July 2012 (n = 1056). Women and their infants were followed up to 6 months postpartum. The present report describes results from the infants born to the mothers enrolled in the study. The study used the trivalent IIV recommended by the World Health Organization for the Southern Hemisphere for both the 2011 and 2012 influenza seasons (A/California/7/2009, A/Victoria/210/2009 and B/Brisbane/60/2008-like virus; VAXIGRIP; Sanofi-Pasteur, Lyon, France) [20, 21], and sterile 0.9% normal saline solution as placebo (1:1).

### Surveillance of Participants

Active surveillance for acute respiratory illness was done by weekly contact of the study participants throughout the study period. Nasopharyngeal aspirates were collected when infants (i) attended the study center for any unsolicited respiratory illness; (ii) were hospitalized for acute cardiopulmonary illness at the single public hospital serving the study population; or (iii) were identified through weekly contacts as having signs or symptoms of respiratory illness. Nasopharyngeal aspirates were collected in universal transport medium (Copan, Brescia, Italy) and transported to the study laboratory where samples were immediately tested by qualitative real-time reverse transcription PCR assay for influenza; the remaining specimens were stored and tested at a later stage for *Bordetella pertussis*, respiratory syncytial virus (RSV), and rhinovirus by PCR.

All-cause hospital admissions were documented and for the current analysis, hospitalizations for ALRI were defined as a discharge with a principal or secondary diagnosis with an *International Classification of Diseases, Tenth Revision* (*ICD-10*) codes of 1 or more of the following: pneumonia (J12–J18), bronchiolitis (J21), or an unspecified acute lower respiratory tract infection (J22). To account for misclassification of congenital pneumonia and other causes of respiratory distress during the newborn period, only hospitalized infants older than 7 days were included in the analyses. The diagnosis of neonatal jaundice, unspecified (P59.9) was analyzed as an outcome that should be unaffected by vaccine exposure. Bacterial cultures from a normally sterile site (eg, cerebrospinal fluid [CSF], blood, or pleural fluid) were performed at the discretion of the attending physician and results were available to the study team.

### Statistical Analysis

All analyses of study outcomes were performed under the principle of intention-to-treat, defined as infants born any time after their mothers’ vaccination. A respiratory pathogen was associated with a hospitalization if it was detected by PCR on a respiratory specimen collected within 2 weeks of the date of hospital admission.

Incidence rates of ALRI hospitalizations were calculated as incidence density using Poisson regression and person-time as denominator; incidence rate ratios were estimated between the IIV group and placebo group. Time-to-event data were censored after the first ALRI hospitalization or at study termination (maximum 175 days of age). Between-group differences in the time to the ALRI episode were compared in survival analyses by means of the log-rank test. The South African influenza seasons were defined using the National Institute for Communicable Diseases surveillance data; the influenza epidemic periods were from 16 May to 6 November 2011 and from 21 May to 14 October 2012 [10, 22]. An exploratory analysis was done restricted to the hospitalizations that occurred within the influenza seasons ±2 weeks (extended influenza season period) to account for possible events that lag in time to influenza virus infection. Proportions were compared by χ^2^ or Fisher exact test and demographic continuous variables by Student *t* test or Mann-Whitney test. Logistic regression was performed to adjust the analysis for sex and low birth weight. *P* values ≤ .05 were considered significant. Study data were collected and managed using Research Electronic Data Capture (REDCap). Analyses were performed using Stata version 13.1 (StataCorp, College Station, Texas).

### Ethical Considerations

The study was approved by the Human Research Ethics Committee of the University of the Witwatersrand (101106), was registered at ClinicalTrials.gov (NCT01306669), and was conducted in accordance with Good Clinical Practice guidelines. Mothers provided written informed consent for themselves and their infants.

## RESULTS

A total of 2049 live births were recorded from pregnant women enrolled in the study, including 1026 to IIV recipients and 1023 to placebo recipients (Table 1). Infants were born a mean of 81 days (range, 1–175 days) after maternal vaccination and followed up for a median of 172 days (interquartile range [IQR], 168–175). The baseline demographics and follow-up period were similar between infants in the IIV and placebo groups (Table 1). Due to the primary objective of the study and when enrollment occurred, most of the follow-up time of the infants on the study was during the periods of extended influenza circulation, with an overall 11201 months of follow-up, of which 6514 months were during the extended influenza seasons. Infants were particularly exposed to influenza virus during the first 90 days of life (5933 months of total follow-up, of which 4743 [80.4%] months were during the extended influenza seasons) compared to during 91–175 days of life (5268 months of total follow-up, of which 1771 [33.6%] months were during the extended influenza seasons).

**Table 1.** Infant Characteristics

Characteristic	IIV (n = 1026)	Placebo (n = 1023)
Girls, No. (%)^a^	484 (47.2)	484 (47.3)
Births <37 wk gestational age, No. (%)	108 (10.5)	96 (9.4)
Mean birth weight, kg (SD)^b^	3.0 (0.5)	3.1 (0.5)
Birth weight <2500 g, No. (%)^b^	133 (13.0)	122 (12.0)
Mean days between maternal vaccination and birth (SD)	81.5 (35.3)	80.6 (35.4)
Median days of follow-up (IQR)	172 (168–175)	172 (168–175)
Deaths, No. (%)	15 (1.5)	21 (2.1)

Abbreviations: IIV, inactivated influenza vaccine; IQR, interquartile range; SD, standard deviation.

^a^Information on sex missing in 1 infant in the IIV group.

^b^Birth weight information missing in 2 infants in the IIV group and 2 infants in the placebo group.

Three hundred fourteen infants had at least 1 hospital admission during the study follow-up period; of these, 151 were born to IIV recipients and 163 to placebo recipients (risk ratio, 0.92 [95% confidence interval {CI}, .75–1.1]; *P* = .42). Fifty-two infants at a median age of 72 days (IQR, 32–131) were hospitalized for physician-diagnosed ALRI during the follow-up period. Only 2 of the hospitalized infants with ALRI were born <28 days after their mothers were vaccinated (18 and 25 days, both to IIV recipients). Of the 52 hospitalized infants, 11.5% were born at <37 weeks of gestational age compared with 9.4% of the 1997 nonhospitalized infants (*P* = .67), 21.2% (11/52) had a birth weight of <2500 g compared with 12.2% (244/1993) of nonhospitalized infants (*P* = .055), and boys were more frequently hospitalized for ALRI (67.3% [35/52]) than girls (*P* = .033).

**Figure 1.**
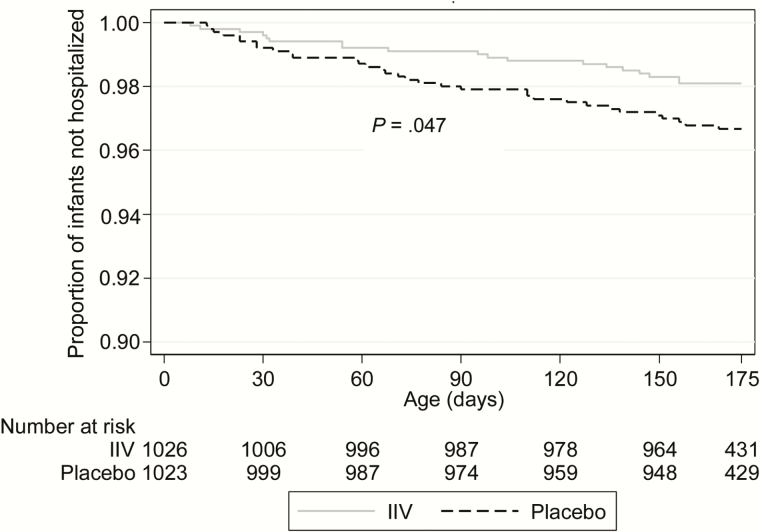
Kaplan-Meier survival curve showing cumulative proportion of infants without lower respiratory infection–associated hospitalizations during the follow-up period by study group. *P* values calculated by log-rank test. Abbreviation: IIV, inactivated influenza vaccine.

Of the 52 hospitalized infants with ALRI, 19 were born to IIV recipients and 33 to placebo recipients for a vaccine efficacy of 43.1% (incidence rate ratio [IRR], 0.57 [95% CI, .32–1.0]; *P* = .050; Table 2 and Figure 1). Time to ALRI hospitalization was longer in the IIV group compared with the placebo group (*P* = .047; Figure 1). Thirty (57.7%) ALRI hospitalizations occurred during the first 90 days of life, 9 in the IIV group (47.4%) and 21 in the placebo group (63.6%). The incidence (per 1000 infant-months) of ALRI hospitalization was lower in infants born to IIV recipients (3.0 [95% CI, 1.6–5.9]) compared to placebo recipients (7.2 [95% CI, 4.7–11.0]) (IRR, 0.43 [95% CI, .19–.93]; *P* = .032) for a vaccine efficacy of 57.5% during the first 90 days of life. In infants >90 days of age, the incidence (per 1000 infant-months) of ALRI hospitalization was similar between the IIV group (3.8 [95% CI, 2.0–7.1]) and placebo group (4.6 [95% CI, 2.6–8.1]) (IRR, 0.83 [95% CI, .36–1.9]; *P* = .66; Table 2). Similar estimates were obtained when restricting the ALRI hospitalizations to those that occurred during the extended influenza seasons (Table 2). Outside the influenza season period, although a similar trend was observed, numbers were too small for further stratification by age group.

**Table 2.** Incidence Rates of Lower Respiratory Infection–Associated Hospitalizations by Study Group

Outcome and Infant Age	IIV		Placebo		Incidence Rate Ratio (95% CI)	*P* Value	Adjusted Incidence Rate Ratio^b^ (95% CI)	Adjusted *P* Value^b^
	No.	Rate (95% CI)^a^	No.	Rate (95% CI)^a^				
Overall study follow-up								
Infants ≤90 d	9	3.0 (1.6–5.9)	21	7.2 (4.7–11.0)	0.43 (.19–.93)	.032	0.42 (.19–.92)	.030
Infants 91–175 d	10	3.8 (2.0–7.1)	12	4.6 (2.6–8.1)	0.83 (.36–1.9)	.66	0.82 (.36–1.9)	.65
Infants ≤175 d	19	3.4 (2.2–5.4)	33	6.0 (4.3–8.5)	0.57 (.32–1.0)	.050	0.56 (.32–.99)	.046
During extended influenza season^c^								
Infants ≤90 d	8	3.4 (1.7–6.9)	15	6.3 (3.8–10.4)	0.55 (.23–1.3)	.17	0.54 (.23–1.3)	.16
Infants 91–175 d	6	6.7 (3.0–14.9)	6	6.9 (3.1–15.4)	0.97 (.31–3.0)	.95	0.98 (.32–3.0)	.97
Infants ≤175 d	14	4.4 (2.6–7.4)	21	6.5 (4.2–10.0)	0.67 (.34–1.3)	.25	0.67 (.34–1.3)	.24
Outside extended influenza season								
Infants ≤175 d	5	2.1 (.88–5.1)	12	5.2 (3.0–9.2)	0.40 (.14–1.1)	.09	0.40 (.14–1.1)	.09

Abbreviations: CI, confidence interval; IIV, inactivated influenza vaccine.

^a^Rates calculated as number of cases per 1000 infant-months, using person time between birth and event or end of study.

^b^Adjusted for birth weight <2500 g and sex.

^c^Extended influenza season was defined as the period between 2 weeks prior to the start of and 2 weeks after end of the epidemic influenza period each year.

The median length of ALRI hospitalization was similar in the IIV group (4 [IQR, 2–8] days) and the placebo group (6 [IQR, 3–8] days; *P* = .64). Forty-four of the ALRI hospitalized infants (84.6%) had a respiratory sample collected during (n = 35) or within 5 days of hospitalization (n = 9) that were tested by PCR for influenza virus, of whom only 1 infant (71 days old in the placebo group) tested positive. Of these respiratory samples, 41 (93.2%) were available and further tested by PCR for *Bordetella pertussis* and RSV and 36 (81.8%) were tested for rhinovirus. *Bordetella pertussis* was identified in 3 infants (2 [11.8%] in the IIV group and 1 [4.2%] in the placebo group), RSV in 12 (4 [23.5%] in the IIV group and 8 [33.3%] in the placebo group; *P* = .73), and rhinovirus in 11 infants (3 [21.4%] in the IIV group and 8 [36.4%] in the placebo group; *P* = .47).

Of the 52 hospitalized ALRI cases, blood cultures were performed in 26 (50%) infants (57.9% of 19 cases in the IIV group and 45.5% of 33 in the placebo group) and CSF cultures in 17 (32.7%) infants (42.1% of the IIV group and 27.3% of the placebo group). No pathogenic bacteria were isolated from the blood or CSF specimens.

One infant in the IIV group who was diagnosed with suspected pertussis associated with pneumonia died in hospital at 61 days of age. Two infants in the placebo group died at home 11 days postdischarge at 84 and 173 days of age. During the first 90 days of life, 93 infants were hospitalized with jaundice; 47 born to IIV recipients and 46 to placebo recipients (IRR, 1.0 [95% CI, .68–1.5]; *P* = .92).

## DISCUSSION

Using our randomized, placebo-controlled clinical trial as a vaccine probe, we demonstrate that vaccination with IIV during pregnancy reduced the risk for all-cause ALRI hospitalization by 57.5% during the first 90 days of life. Notably, this observation was independent of identifying influenza virus among the ALRI cases. This suggests that the benefits of protecting against influenza virus infection during early infancy might extend beyond protecting only against influenza-confirmed illness. While the paucity of laboratory-confirmed influenza hospitalizations may be partly explained by inadequate sample or imperfect test sensitivity, it is also likely that the influenza virus may initiate a causal chain of events and no longer be present or detectable at the time of hospitalization. For example, a primary influenza virus infection, including possibly subclinical or mild infection, may increase susceptibility to new bacterial nasopharyngeal acquisition, as well as increase density of present colonizing bacteria, with disease from these bacteria only manifesting a few weeks later and beyond when influenza virus shedding has cased [3, 23]. This hypothesis is corroborated by a previous report from a randomized clinical trial (RCT) of pneumococcal conjugate vaccine, in which was shown that by protecting against pneumococcal disease, there was also a decrease in hospitalizations for influenza, RSV, human metapneumovirus, and polyomavirus-associated ALRI [24–26]. The current data suggest that protecting against these virus-associated infections could conversely protect against bacterial infections. The failure to identify bacterial etiology among our cases is not surprising, considering the lack of a sensitive diagnostic tool to diagnose bacterial pneumonia, including the sensitivity of blood culture only being <5% for diagnosing bacterial pneumonia in children [27].

Epidemiological evidence from influenza epidemics and animal challenge models have demonstrated that influenza virus infection can enhance the susceptibility to infection with bacteria [28, 29], including *Streptococcus pneumoniae*, *Haemophilus influenzae*, and *Staphylococcus aureus* [23, 30–32]. In a South African study among patients hospitalized with ALRI, infection with influenza virus was associated with *S. pneumoniae* colonization and patients with a respiratory virus coinfection had significantly higher colonization densities and, consecutively, increased risk of invasive pneumococcal pneumonia [33]. The co-pathogenesis between influenza and superinfecting bacteria is complex and multifactorial, with sequential infections being challenging to correctly diagnose if the first pathogen has cleared by the time the patient presents with secondary bacterial complications [3]. In the case of *S. pneumoniae*, a new serotype acquisition is associated with increased susceptibility to developing disease up until 2 months later, by which time influenza shedding may no longer be present [8]. Likewise, it is biologically plausible that a preceding influenza infection could lead to airway changes that predispose to more severe disease with subsequent infections as well.

Using a vaccine-probe approach, we estimated that for every 1000 pregnant women vaccinated with influenza vaccine, 4 ALRI hospitalizations could be prevented in infants <3 months of age. Two-thirds of the ALRI hospitalizations in the placebo group occurred during the first 3 months of life, corroborating that very young infants are at increased risk of severe pneumonia [6, 34]. The observation from our trial that even in older infants, who had less exposure to the influenza season, >50% of the hospitalizations occurred during the periods of influenza circulation supports the temporal association of influenza virus infections and ALRI hospitalizations.

This is the first RCT, to our knowledge, which has measured the efficacy of influenza vaccination during pregnancy on severe respiratory outcomes in infants. Two earlier large retrospective cohort studies from the United States did not find an association of influenza vaccination during pregnancy and the rates of acute respiratory illness among their infants [14, 17]. These studies were, however, limited either by the study design or the absolute rates of hospitalization in the infants being very low, limiting the statistical power [14, 17]. In another prospective cohort study in the White Mountain and Navajo reservations in the United States, Eick et al reported a nonsignificant association between maternal influenza vaccination and influenza-like illness in the infants; however, when the outcome was restricted to influenza-like illness requiring hospitalization, a 39% reduction in risk (risk ratio, 0.61 [95% CI, .45–.84]) was found for infants born to IIV-vaccinated compared with unvaccinated mothers [16]. In accordance, although we demonstrate here the efficacy of IIV vaccination during pregnancy against infant ALRI hospitalization, we did not observe a significant effect on medically attended outpatient visits at our study clinic [10]. Furthermore, a recent retrospective cohort study from Australia also described that influenza vaccination during pregnancy was associated with a 25% reduction (adjusted hazard ratio, 0.75 [95% CI, .56–.99]) in hospitalizations for any acute respiratory illness in infants <6 months of age during influenza season [19]. Additionally, 3 other observational studies found that influenza vaccination during pregnancy was associated with a 48%–91% reduced risk of laboratory-confirmed influenza hospitalizations in infants <6 months of age [13, 15, 18].

The lack of tools to adequately investigate for bacterial pneumonia in infants and the fact that chest radiograph information was not available for this study to use as a proxy for bacterial pneumonia, albeit it being a more specific than sensitive marker for pneumococcal pneumonia, are limitations of our study [27]. Furthermore, not all infants were investigated for evidence of influenza virus infection at the time of hospitalization.

As influenza virus infection can be asymptomatic and the virus might be undetectable by the time the patient presents for care, even if the virus was part of the causal pathway of infection, we used the randomized placebo-controlled design of our trial as a vaccine probe to delineate the effect of maternal influenza vaccination on all-cause ALRI hospitalization in infants [35]. This type of analysis helps to more comprehensively describe the disease burden than simple etiologic studies that may underestimate the real impact of influenza infection.

## Notes

***Acknowledgments.*** The authors thank all the study participants, the staff of the Departments of Obstetrics, Neonatology, and Paediatrics at Chris Hani Baragwanath Academic hospital, Soweto, South Africa, for their dedication to their patients, including our trial participants; and the study midwives, nurses, laboratory staff, counselors, and data capturers. We thank Niteen Wairagkar, program officer acting on behalf of the Bill & Melinda Gates Foundation, David Moore from Respiratory and Meningeal Pathogens Research Unit, and Jorge Vidal from Emory University.

***Financial support.*** This study was supported by the Bill & Melinda Gates Foundation (grant number OPP1002747). There was also partial support from the South African Research Chairs Initiative of the Department of Science and Technology and National Research Foundation in Vaccine Preventable Diseases; and the Medical Research Council Respiratory and Meningeal Pathogens Research Unit.

***Potential conflicts of interest.*** All authors: No reported conflicts of interest. All authors have submitted the ICMJE Form for Disclosure of Potential Conflicts of Interest. Conflicts that the editors consider relevant to the content of the manuscript have been disclosed.
